# The Role of Amygdala in Patients With Euthymic Bipolar Disorder During Resting State

**DOI:** 10.3389/fpsyt.2018.00445

**Published:** 2018-09-19

**Authors:** Gaizhi Li, Penghong Liu, Elissar Andari, Aixia Zhang, Kerang Zhang

**Affiliations:** ^1^Shanxi Medical University, The First Hospital of Shanxi Medical University, Taiyuan, China; ^2^Department of Psychiatry and Behavioral Sciences, Center for Translational Social Neuroscience, Silvio O. Conte Center for Oxytocin and Social Cognition, Yerkes National Primate Research Center, Emory University School of Medicine, Atlanta, GA, United States

**Keywords:** bipolar disorder, euthymia, amygdala, resting state fMRI, fALFF

## Abstract

The current study aims to explore the functional changes of the amygdala in patients with euthymic Bipolar Disorder (BD) using resting state fMRI (rs-fMRI). Twenty-one euthymic patients with bipolar disorder and 28 healthy controls participated in this study. Two of the euthymic patients with BD and three of the healthy controls were excluded due to excessive head motion. We found that patients with euthymia (38.79 ± 12.03) show higher fALFF (fractional Amplitude of low-frequency fluctuation) value of the amygdala (*t* = 2.076, *P* = 0.044), and lower functional connectivity between the amygdala and supplementary motor area (*p* < 0.01, GRF corrected) than healthy controls (33.40 ± 8.21). However, euthymic patients did not show a differential activity in ReHo (Regional Homogeneity) and gray matter of the amygdala region as compared to healthy controls. Thus, despite the absence of clinical symptoms in euthymic patients with BD, the amygdala functional activity and its connectivity to other brain regions remain altered. Further investigation of negative emotions and social functioning in euthymic patients with BD are needed and can help pave the way for a better understanding of BD psychopathology.

## Introduction

Bipolar disorder (BD) is a chronic psychiatric disorder that is characterized by recurring manic or hypomanic episodes and additional episodes of depression usually separated by periods of euthymia (1). The disorder is characterized by dramatic shifts in mood that can have negative repercussions on cognitive abilities and quality of life (2). Based on diagnostic interview data from National Comorbidity Survey Replication, an estimate of 4.4% of U.S. adults experience BD at some time in their lives (3), with a prevalence of 1.0% (0.6%) for BD-I, 1.1% (0.8%) for BD-II, and 2.4% (1.4%) for subthreshold BD in general population according to the report of National Comorbidity Survey of US (4).

A large body of evidence implicates dysfunction in brain networks related to emotion regulation in BD (5). A common theory is that these patients show a hypoactivation in areas related to top-down control (such as the ventrolateral prefrontal cortex), and a hyperactivation in areas related to affective salience (such as the amygdala) (6). This imbalance between the two systems can lead to episodes of mania and depression. In particular, BD is consistently associated with deficiency in the amygdala activity and in global network communication across several brain regions (7). Patients with BD with depressive episodes show lower resting state functional connectivity (rsFC) between areas involved in emotion regulation such as the insula and prefrontal cortex compared to patients with major depressive disorders and to healthy controls (8). Other studies showed a decreased rsFC between areas involved in emotional regulation such as the amygdala and frontal cortex (as well as ventral PFC) as compared to HC (9, 10). It has been shown significant differences in rsFC between patients with depression and patients with manic states while both having BD (9). For instance, in a very interesting study that aimed to dissociate neural correlates of manic symptoms from depressive symptoms in BD, authors identified hyperconnectivity in a network involving the amygdala that is related to manic symptoms and hyperconnectivity in a network involving OFC that is related to depressive symptoms (7). These results suggest that patients with BD have significant alterations in rsFC between areas involved in emotional and cognitive processes and that these disrupted networks can vary based on the type of symptoms that these patients display.

Euthymia is usually described as a stable mental health state in patients with BD that is free from depression and manic episodes. However, despite the absence of clinical symptoms, these patients with euthymic BD report challenges and difficulties in adaptive skills and functioning. They do not recover completely and show difficulties in attaining their premorbid functioning (11). However, much less is known about the euthymic period and its underlying mechanisms. Some neuroimaging studies have shown abnormal BOLD activity in the amygdala, orbitofrontal cortex (OFC), and medial prefrontal cortex (mPFC) in response to emotional stimuli during remission (12, 13). More specifically, the BOLD signal of the amygdala was found abnormally elevated in response to fearful facial expressions in individuals with euthymic-BD (14, 15). These abnormalities could underlie deficits in affective and cognitive integration in bipolar I euthymia.

In line with these findings, there is also an elevated functional connectivity (FC) between right amygdala and OFC in response to sad faces, in comparison to healthy controls (HCs) (16), suggesting a higher degree of anxiety or emotion dysregulation in these patients. However, these findings were not selective to the euthymic period, instead were also observed in depressive episodes of these patients (17–20). Thus, more research is needed to better characterize the brain circuitry and, in particular, the role of amygdala in these individuals.

Here, we aim to investigate brain functional connectivity and the intensity of amygdala activity, which was found to play an important role in BD, in patients with euthymic-BD during resting state. Understanding the neural correlates of the euthymic period can provide crucial knowledge on brain plasticity and potential biomarkers for clinical recovery for patients with BD.

We hypothesize that despite the absence of clinical symptoms of depression or mania, these patients with euthymic-BD will show altered amygdala functional activity and connectivity with other brain regions as compared to HCs.

## Materials and methods

### Participants

Patients were recruited at the First Hospital of Shanxi Medical University between Sep-1-2015 and Sep-1-2016. The diagnosis (BD I/II) was confirmed by the psychiatrist of the study (KZ or Pr. Zhang) using SCID II and following DSM-IV criteria. After the BD diagnosis, patients were asked to come back for 4 follow-up clinical visits during a period of 4 weeks (one visit every week). During these follow-up visits, KZ interviewed the patient and assessed manic and depressive symptoms using the HAMD (Hamilton Depression Rating Scale-17 items) and YMRS (Young Mania Rating Scale) scales (21, 22). Patients were determined as euthymic if the scores on the HAMD were less than 7 and on the YMRS as less than 5 during the 4 consecutive visits. Upon determination of euthymia, patients were asked to come back for follow-up regular visits every 2 weeks for research purposes. Thus, euthymia was confirmed based on clinical interviews and HAMD/YMRS scores.

The inclusion criteria for the euthymic group consisted of the following criteria: (1) age: between 18 and 60 years; (2) depression as previous episode; (3) the duration of euthymia is at least 30 days; (4) HAMD score <7; (5) YMRS score <5. The exclusion criteria consisted of: (1) any history of neurological diseases, other physical diseases and presence of comorbidities of other disorders; (2) any other mental disorders, e.g., schizophrenia, schizoaffective disorder, substance use disorder, OCD, panic disorder, generalized anxiety disorder, social phobia, post-traumatic stress disorder, Axis II personality disorders, or mental retardation; (3) Pregnancy or breastfeeding. Axis II personality disorders were excluded by using Structured Clinical Interview for DSM-IV and Axis II Personality Disorders (SCID-II) Interview (23). Patients were also screened for MRI safety.

HCs were recruited through local advertisement in Taiyuan, China. Criteria of inclusion consisted of: (i) aged 18–60 years; (ii) no history of psychiatric illness or substance abuse/dependence; (iii) no family history of major psychiatric or neurological illness in first degree relatives; (iv) no psychotropic or prescribed medications; (v) no use of alcohol in the past week and (vi) no serious medical or neurological illness. Exclusion criteria included: (i) pregnancy or breast feeding and (ii) metallic implants or other MRI contraindications. None of the HCs had a history of any neuropsychiatric disorders or personality disorders. The HCs were screened by a M.D.-level student using the HAMD-17, YMRS, and SCID-II. Two of the euthymic patients with BD and three of the healthy controls were excluded during data analysis due to excessive head motion. The analysis was conducted on the MRI data of 19 euthymia and 25 HCs. The male to female ratio in the euthymia group is 10:9 and 15:10 in the HC group.

This study protocol was approved by the Institutional Review Board of The First Hospital of Shanxi Medical University in China. Written informed consent was obtained from all the participants.

### Clinical questionnaires

Hamilton Depression Rating Scale (HAM-D) was administered to assess severity of depressive symptoms in patients (21). The Young Mania Rating Scale (YMRS) was administered to assess severity of mania/hypomania (22).

### MRI data acquisition and processing

MRI scans were performed in a 3.0 T Trio Siemens System at Shanxi Provincial People's Hospital in China using the following parameters: repetition time (TR) = 2300 ms, effective echo times (TE) 2.95 = ms, thickness/skip = 1.2/0.6 mm, sagittal slices = 160, FOV = 225 × 240 mm, matrix = 240 × 256 mm, FA = 90°, 160 volumes.

Subjects were asked to rest quietly for 6 min total and to close their eyes during rs-fMRI data acquisition. We used a gradient-echo single-shot echo planar imaging (EPI) to acquire rs-fMRI image volumes. We acquired 212 three-dimensional image volumes. Each of these volumes comprises 16 noncontiguous axial sections parallel to the inter commissural plane, with the following parameters: TR = 2,000 ms; TE 30 ms; section thickness 3 mm; slices = 32; field of view (FOV) = 240 × 240 mm^2^, matrix = 64 × 64 mm^2^, flip angle (FA) = 90°.

VBM 8 was used for data preprocessing of T1. The preprocessing steps included: estimate and write, display one slice for all images, check sample homogeneity using covariance. The images were then smoothed with an 8-mm full width at half-maximum (FWHM) Gaussian kernel.

Rs-fMRI data was preprocessed using DPABI (Data Processing Assistant for Resting State fMRI) by Yan et al. (24). The preprocessing included: a removal of the first 10 time points, slicing timing, realignment, normalization, smoothing with a 6-mm FWMH Gaussian kernel and nuisance covariates regression. Data with head motion of more than 2.5 mm or an angular rotation of greater than 2.5° in any direction was excluded from the analysis. We conducted regional homogeneity analysis (ReHo) to study functional synchronization (25, 26). We also conducted fALFF (fractional Amplitude of low-frequency fluctuation) analysis to study the intensity of regional spontaneous brain activity (27). We used the software REST 1.8 (28) to calculate ReHo and fALFF scores of the amygdala. The functional connectivity between the amygdala and the whole brain were analyzed using seed-based functional connectivity by DPABI.

### Statistical analysis

Clinical data was analyzed using SPSS 17.0. Structure MRI data was analyzed using SPM 8. Independent two sample *t*-tests were used to compare rs-fMRI data between BD and HC groups using REST 1.8. The *p*-value for MRI data was set as <0.01 (GRF corrected for multiple comparisons, with voxel *p* < 0.01).

## Results

There was no significant difference in age between patients with euthymia and HCs (38.79 ± 12.03; 33.10 ± 8.21, respectively; *t* = 1.679, *P* = 0.104). All the patients were taking lamotrigine as medication (Table 1).

**Table 1:** Demographic data of the euthymic-BD group and HC group.

	**Euthymic-BD group (*n* = 19)**	**HC group (*n* = 25)**	***t/x^2^***	***P***
Age	38.79 ± 12.03	33.40 ± 8.21	1.679	0.104
Gender (male:female)	10:9	15:10	0.239	0.625
BD I/II	6/13	–		
Education years	16.71 ± 4.65	18.29 ± 3.10	−1.227	0.231
Work/not work	14/5	25/0	–	–
Age of illness onset	34.84 ± 10.86	–	–	–
Illness duration	3.95 ± 3.30	–	–	–
Episodes	3.73 ± 1.37	–	–	–
Time in euthymia (months)	5.63 ± 3.00	–	–	–
Medication	19 lamotrigine		–	–
HAMD	4.22 ± 1.69	–	–	–
YMRS	2.63 ± 1.49	–	–	–

The analysis conducted on brain volume for both groups revealed no significant differences in gray matter in the amygdala region. We also did not find any difference between the two groups in terms of ReHo in the amygdala region during resting state.

**Figure 1:**
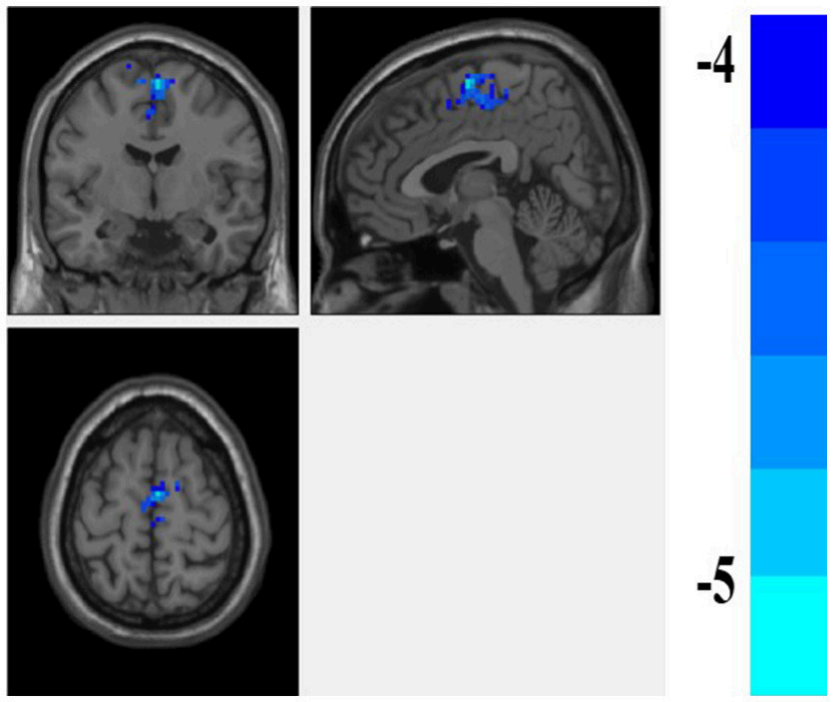
The difference of FC of the amygdala between the euthymic BD and HC groups. the FC of amygdale and supplementary motor area (SMA) is lower in the patients with euthymic BD than the HCs.

We found that fALFF scores were significantly higher in the amygdala region in patients with euthymia as compared to HC group (*t* = 2.076, *P* = 0.044) during resting state (see Table 2). We also found that the functional connectivity between the amygdala, bilateral supplementary motor area (SMA), and left Paracentral lobule is significantly lower in patients than in HCs (Table 3 and Figure 1).

**Table 2:** The structural and functional difference of Amygdala between the euthymic BD and HC groups.

	**Euthymic-BD group (*n* = 19)**	**HC group (*n* = 25)**	***t/x^2^***	***P***
ALFF	0.891 ± 0.216	0.806 ± 0.132	1.600	0.117
fALFF	0.895 ± 0.051	0.859 ± 0.061	2.076	0.044
ReHo	0.765 ± 0.073	0.742 ± 0.074	1.004	0.321
GM volume	0.792 ± 0.059	0.832 ± 0.081	−1.759	0.086

**Table 3:** The FC of the amygdala between the euthymic BD and HC groups.

**Voxels**	**L/R**	**Brain areas**	**BA**	**MNI**			**Peak intensity**
				***x***	***y***	***z***	
82	R	SMA	6	3	−6	63	−4.9728
42	L	SMA					
15	L	Paracentral lobule					

## Discussion

Here, we found that fALFF score in the amygdala region was significantly higher in patients with euthymia than in HCs. We also found that the rs-FC of amygdala with SMA and paracentral lobule was lower in these patients as compared to HCs.

The fALFF score is a sensitive index of resting state brain activity (29). Higher fALFF score in euthymia is an indicator of an abnormal brain activity during resting state. These results are in line with other studies in the literature showing an increased activity in the amygdala region in response to negative emotional stimuli in these patients, as compared to HCs (30–32). Amygdala activity has been shown to be related to fear and negative valence in the literature. A predominant higher activity in these regions can be an indicator of an emotional dysregulation in these euthymic patients. The increased fALFF score is also consistent with the known hyperconnectivity in networks related to amygdala in BD and in BD with remitted episodes (6).

Analysis of functional connectivity can be an important indicator of the correlations of neural activity among different brain regions and different networks. It is defined by “the temporal correlation of neurophysiological indexes measured by low frequency BOLD—fMRI signals.” In this study, we found a significant lower FC between the amygdala and sensory-motor regions (SMA and paracentral lobule) in patients with euthymia as compared to healthy controls. These results contradict the findings of a previous study that shows an increased functional connectivity between the amygdala and SMA in patients with BD under mania and euthymic states (33). In addition, other studies have examined the functional connectivity of euthymic period of patients with BD and found an increased connectivity between other brain networks. For example, Lois et al. found an increased functional connectivity between the meso/paralimbic and the right frontoparietal network (34). Torris et al. reported a hyperconnectivity between the right amygdala and the right vlPFC relative to healthy subjects (35). Hence, despite the absence of major clinical symptoms in the euthymic group, these patients still have differential brain activity and functional connectivity as compared to HCs. The main focus of the previous studies was the FC between temporal and frontal areas in these patients. It would be crucial to examine the functional connectivity between the amygdala and sensory-motor areas in future studies. More replications are needed in order to better characterize the deficit in this particular network. Indeed, previous studies reported a reduced SMA volume in children with BD (36). Aron et al. (37) proposed that pre-SMA, DLPFC, and striatal regions comprise a network mediating motor and cognitive inhibition. These functions are impaired in BD and in unaffected BD relatives (38–40). SMA is a region that mediate the control and execution of motor actions, a finding that could be related to increased self-consciousnessness. A recent study (41) showed that self-consciousness was predicted by a weaker functional coupling of the right amygdala and the SMA region. It is possible that these patients suffer from self-consciousness, an area that is not very well investigated in BD. Hence, it will be interesting to investigate the role of neuroticism and negative emotions in BD and how is this related to rsFC between amygdala and SMA region.

Volumetric analysis on the gray matter in the amygdala region reveals no significant difference between the two groups. The results show a non-significant trend for a lower gray matter volume in euthymic group (*P* = 0.086). It is possible that these results will reach a significant threshold with a bigger sample size. Other studies have reported a smaller gray matter volume in BD patients with depression episodes (42). More replications with a larger sample size are needed to better characterize the brain volume deficits in amygdala region in patients with BD and during remission. It is possible that gray matter changes are less plastic and are not yet recovered in some of the patients during remission following depression and mania symptoms. An alternative explanation could be that there are no significant differences between euthymic patients and HCs in terms of brain volume in amygdala brain region. Other authors have found no significant volumetric differences between patients who recently remitted from their first manic episodes and healthy volunteers (43). A preserved amygdala gray matter can be also a biomarker of a promising recovery in terms of clinical symptoms as it has been shown an increased gray matter volume in this region in patients who are resistant to treatment and remain having depression symptoms (44).

## Limitation

One of the limitations of the study is the small sample size. A larger sample is necessary to show gray matter differences in the amygdala region. Another limitation is the sole inclusion of euthymic patients and HCs. Future studies should also include BD patients with depression and mania episodes in addition to patients with euthymia, in order to investigate the brain mechanisms of BD. Investigating the neural correlates and the role of amygdala in relation to the number of episodes, the number of manic and depressive episodes, the chronicity of BD, duration of illness, and the clinical progression is needed. Unfortunately, we were not able to correlate the fMRI results with the previous episodes of depression and mania as we did not have data on the type and scores of all the previous episodes that were encountered in these patients. The focus of the study design was based on euthymia and on the stability of mood symptoms.

Future longitudinal studies in patients with BD would be crucial to better understand the neural mechanisms and pathophysiology of the progression of BD and the switch between clinical depression and remission. This would help unraveling biological targets for future treatments for bipolar depression.

## Conclusion

Our findings indicated an abnormal amygdala activity during resting state in euthymic patients depicted by a higher fALFF score and lower FC between the amygdala and SMA. Our results may provide useful information to further understand the neurobiological basis of BD.

## Author contributions

GL analyzed the neuroimaging data and wrote the manuscript. KZ designed this study. EA wrote and revised the manuscript. PL and AZ collected the data.

### Conflict of interest statement

The authors declare that the research was conducted in the absence of any commercial or financial relationships that could be construed as a potential conflict of interest.
